# Validation and Verification of High-Fidelity Simulations of Thoracic Stent-Graft Implantation

**DOI:** 10.1007/s10439-022-03014-y

**Published:** 2022-07-19

**Authors:** Anna Ramella, Francesco Migliavacca, Jose Felix Rodriguez Matas, Frederic Heim, Francesca Dedola, Stefania Marconi, Michele Conti, Sara Allievi, Tim J. Mandigers, Daniele Bissacco, Maurizio Domanin, Santi Trimarchi, Giulia Luraghi

**Affiliations:** 1 Computational Biomechanics Laboratory – LaBS, Department of Chemistry, Materials and Chemical Engineering ‘Giulio Natta’, Politecnico di Milano, Piazza L. da Vinci 32, 20133 Milan, Italy; 2 Laboratoire de Physique et Mécanique des Textiles, Université de Haute-Alsace, 11 rue Alfred Werner, 68093 Mulhouse, France; 3 Clinical and Community Sciences Department, Università degli Studi di Milano, Via della Commenda 19, 20122 Milan, Italy; 4 Department of Civil Engineering and Architecture (DICAr), University of Pavia, Via Ferrata 3, 27100 Pavia, Italy; 5 Unit of Vascular Surgery, I.R.C.C.S. Fondazione Cà Granda Policlinico Milano, Via Francesco Sforza 35, Milan, Italy

**Keywords:** Endograft, TEVAR, Finite element method, Numerical model

## Abstract

Thoracic Endovascular Aortic Repair (TEVAR) is the preferred treatment option for thoracic aortic pathologies and consists of inserting a self-expandable stent-graft into the pathological region to restore the lumen. Computational models play a significant role in procedural planning and must be reliable. For this reason, in this work, high-fidelity Finite Element (FE) simulations are developed to model thoracic stent-grafts. Experimental crimp/release tests are performed to calibrate stent-grafts material parameters. Stent pre-stress is included in the stent-graft model. A new methodology for replicating device insertion and deployment with explicit FE simulations is proposed. To validate this simulation, the stent-graft is experimentally released into a 3D rigid aortic phantom with physiological anatomy and inspected in a computed tomography (CT) scan at different time points during deployment with an ad-hoc set-up. A verification analysis of the adopted modeling features compared to the literature is performed. With the proposed methodology the error with respect to the CT is on average 0.92 ± 0.64%, while it is higher when literature models are adopted (on average 4.77 ± 1.83%). The presented FE tool is versatile and customizable for different commercial devices and applicable to patient-specific analyses.

## Introduction

Thoracic Endovascular Aortic Repair (TEVAR) is a minimally invasive technique to treat thoracic aortic pathologies. It has become the most adopted treatment option since the FDA approval of the first endograft in 2005.^15,27^ The procedure consists of placing a self-expandable stent-graft into the pathological region through a catheter to recreate a physiological-like lumen. The endograft is composed of a fabric graft component attached to a metallic stent, which gives structural support to both the graft and the treated aorta.^35^

Computational models are one of the most widely used tools to investigate the TEVAR procedure since they play a significant role either in supporting pre-operative planning and understanding the device performance. Patient-specific simulations can be performed to improve the device development and optimize the procedure.^39^ However, if used for clinical applications, numerical models must be reliable, thus the verification and validation (V&V) process is of foremost importance. To this aim, in 2018, the American Society of Mechanical Engineering (ASME) introduced its first V&V standard for specific applications to medical devices for establishing the credibility needed to support the use of a computational model.^2^ Verification and validation are two different concepts generally used together to demonstrate the credibility of an *in-silico* model. The verification process is referred to the process of determining if the computational model – together with the code used for its implementation – is sufficiently accurate to reproduce the underlying mathematical model. The validation is the process of determining if the mathematical model is accurate in representing the interested physical scenario.^1,28,40^

In the context of endograft numerical simulations, the recent literature involves several finite element (FE) structural studies regarding stent-graft modeling by adopting different strategies. Regarding the stent mesh, either 1D beam elements^11,9,13,21,30,29^ or 3D hexahedral elements^5,14,18,19,32,33^ are adopted; the graft is discretized using 2D triangular or quadrangular shell elements^9,13,21,30^ or membrane elements.^5,11,18,19,32,33^ Regarding the material properties, the graft fabric is modelled as a linear elastic material in most cases or as a linearized orthotropic linear elastic material.^12^ For the stent component, some studies simplify the superelastic Nitinol behavior with a linear elastic material model with Young’s modulus equal to the austenitic one.^13,16,21,29^ In addition, a few works introduce the pre-stress field in the unloaded stent component.^7,11,17,19,34^

This study aims at developing high-fidelity FE simulations to virtually reproduce the TEVAR procedure. Experimental tests are used to add V&V evidence in the state-of-the-art on the TEVAR modeling. In particular, it includes (i) calibration of stent and graft materials based on experimental data; (ii) validation of the stent-graft model with experimental data; (iii) deployment simulation of the device into an idealized aorta; (iv) validation of the deployment simulation by comparing the *in-silico* analysis with real device configurations reconstructed from CT images performed on an endograft released in a mock aorta; (v) sensitivity study on different modelling choices to compare the proposed TEVAR model with those found in the literature.

## Materials and Methods

For the purpose of the study, three models of the commercially available Valiant™ thoracic aortic stent graft with the Captivia™ delivery system (Valiant Captivia) (Medtronic, MN, USA) were created with SolidWorks (Dassault Systèmes SOLIDWORKS Corp., MA, USA) from measurements directly taken on the device with a calliper. Sizes 30 × 30 × 150 Closed-Web (device A), 46 × 46 × 150 Free-Flo (device B) and 34 × 34 × 200 Free-Flo (device C) were generated (Figure e 1a).

**Figure 1:**
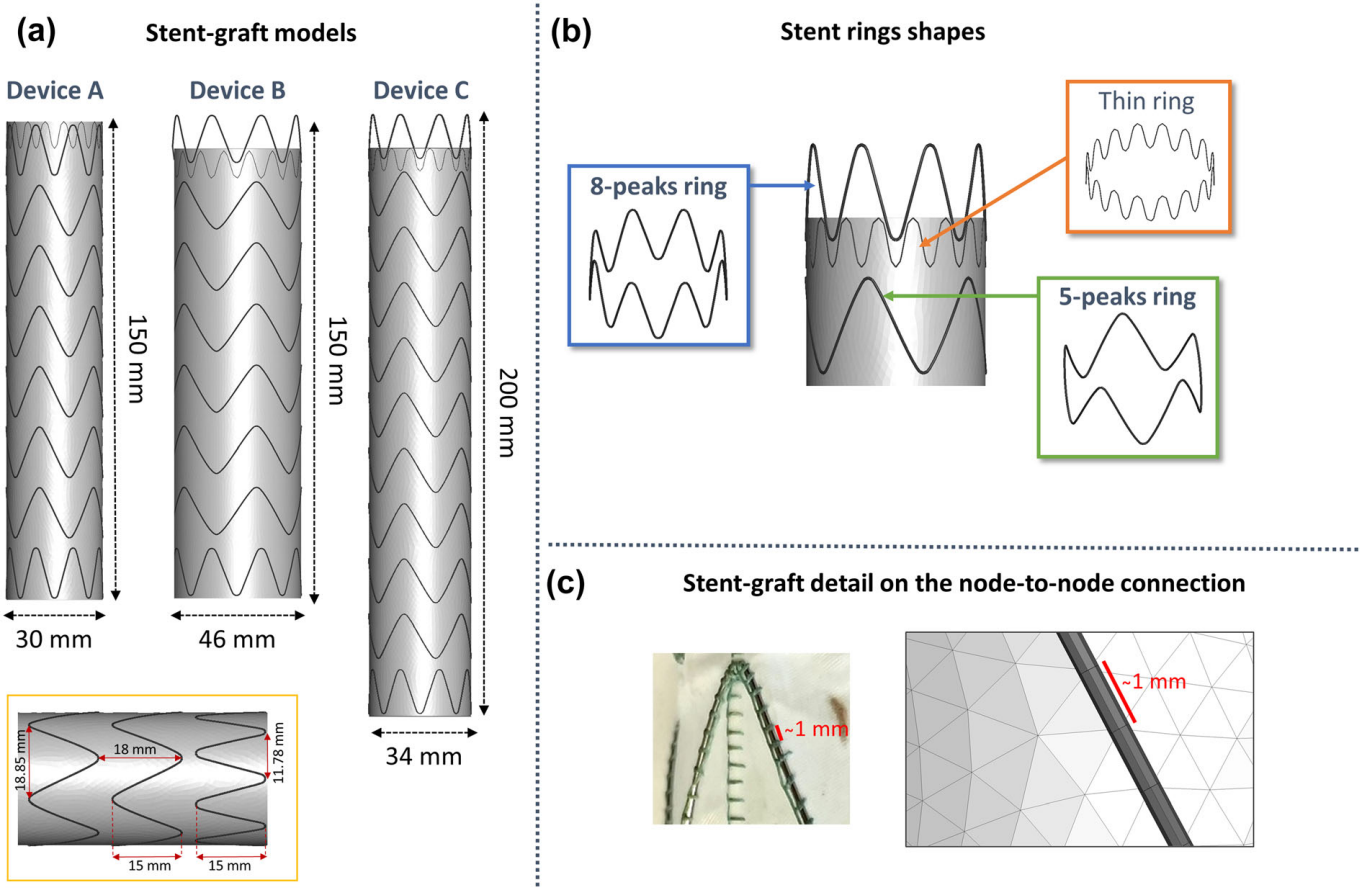
(a) Geometries of the three reconstructed commercial stent-graft used in this study; (b) 8-peaks ring, 5-peaks ring and thin ring which composed the stents; (C) Detail of the graft mesh with respect to the stent one.

The stents are composed of a combination of 8-peaks and 5-peaks rings with a circular cross-section of 0.5 mm and a proximal thin ring with a circular cross-section of 0.2 mm (Figures 1A and 1B). The graft has a thickness of 0.1 mm. Table 1 sums up the device models.

**Table 1:** Summary of the three stent-graft geometries.

Stent-Graft	Diameter	Length	Configuration
Device A	30 mm	150 mm	Closed-Web Straight
Device B	46 mm	150 mm	Free-Flo Straight
Device C	34 mm	200 mm	Free-Flo Straight

The grids in this study were created with ANSA Pre Processor v22.0 (BETA CAE System, Switzerland); finite element simulations were performed on 20 CPUs of an Intel Xeon64 with 120 GB of RAM using the commercial explicit finite element solver LS-DYNA 971 Release 13.0 (ANSYS, Canonsburg, PA, USA). The post-processing analysis was done with META Post Processor v22.0 (BETA CAE System, Switzerland).

### Material Models

Different material models with sometimes discordant values are reported in the literature, the reason why a proper characterization and calibration of the material models were needed.

#### Stent

**Figure 2:**
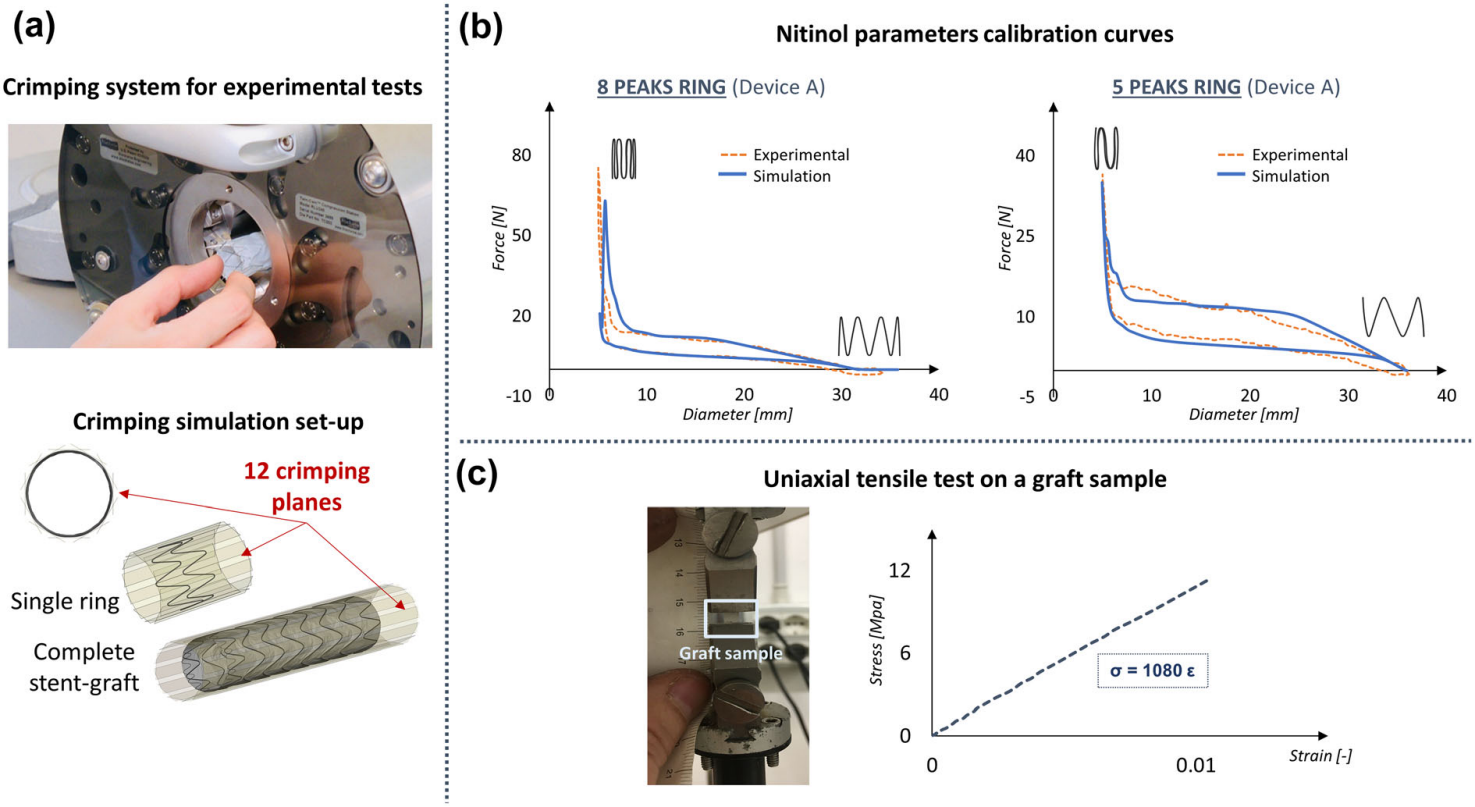
(A) Crimping system for the experimental tests and crimping simulation set-up for the single ring and complete stent-graft model; (B) Calibration of Nitinol parameters curves for the 8-peaks and 5-peaks ring of device A; (C) Uniaxial tensile test of a PET graft sample.

Single stent rings were removed from devices A and B after eliminating the suture points and then subjected to crimping/release. In particular, the removal of the sutures between the stent and graft unveiled that the stent sutured configuration is not stress-free: the zero-stress stent diameter is measured to be higher than the sutured one (+ 17%). Thus, to correctly model and calibrate the Nitinol material, it is necessary to refer to the stress-free configuration.^8^ Crimping and release experimental tests were carried out at 37 °C ± 2 °C (dry air) by using the Blockwise Crimper system (Blockwise Engineering LLC, AZ, USA) (Figure 2A). Starting from the initial configuration, each ring was crimped down to 5 mm and then released back to the initial diameter. The radial force versus diameter history curves were obtained.

From the numerical side, after performing a mesh convergence analysis (reported in the Appendix), each stent ring was discretized with 2 nodes (linear) beam elements with Hughes Liu formulation and 2 × 2 gauss nodes in the circular cross-ection.^22^ In particular, the convergent meshes counted 240 and 160 elements for the 8-peaks and 5-peaks respectively, with an average element size of 1 mm. The stent was modelled adopting the superelastic Nitinol formulation. This model describes the superelastic response present in shape-memory alloys following the description proposed by Auricchio and Taylor,^6^ where the material has the ability to undergo large deformations with a full recovery during loading–unloading cycles. The crimping/release system was reproduced by imposing on 12 rigid planes placed around the stent strut displacement boundary conditions until the 5 mm stent diameter was reached (Figure 2A). Penalty contacts between the planes and stent ring were introduced with a friction value of 0.3 (defined with a sensitivity analysis as reported in the Appendix). Also, a penalty self-contact was introduced to prevent penetration between the stent ring elements. The radial force from the simulations was compared with the experiments. The simulation radial force was the one generated by the contact between the stent and planes. In particular, 12 forces were generated, one for each plane, and the global radial force was the sum of the 12 contributions. After a proper sensitivity analysis (reported in the Appendix), a mass proportional damping factor of 1 s^−1^ was adopted to achieve stability without limiting the maximum timestep (set to 0.001 ms with mass-scaling technique).

For the material calibration process, the Nitinol parameters were gradually adjusted to fit the experimental curve starting from literature parameter values taken from Kleinstreuer *et al.*.^20^ In particular, the Nitinol was calibrated starting from the 8-peaks and 5-peaks rings of device A (Figure 2b). The final Nitinol material properties are reported in Table 2. 8-peaks ring from device B was used to validate the Nitinol parameters.

**Table 2:** Nitinol material parameters after calibration.

Austenite Young’s Modulus	E_A_	57,500 MPa
Austenite Poisson’s Ratio	ν	0.3
Martensite Young’s Modulus	E_M_	47,800 MPa
Martensite Poisson’s Ratio	ν	0.3
Transformation Strain	ε	0.063
Start of Transformation Loading	σ^S^_L_	550 MPa
End of Transformation Loading	σ^E^_L_	620 MPa
Start of Transformation Unloading	σ^S^_U_	450 MPa
End of Transformation Unloading	σ^E^_U_	250 MPa
Start of transformation stress in compression	α	0.0279

Parameters used to model Nitinol are: Austenite and Martensite Poisson’s ratio (ν); elastic modulus of Austenite (E_A_); starting value for the forward phase transformation (conversion of austenite into martensite) (σ^S^_L_); final value for the forward phase transformation (σ^E^_L_); starting value for the reverse phase transformation (conversion of martensite into austenite) (σ^S^_U_); final value for the reverse phase transformation (σ^E^_U_); maximum residual strain (ε), elastic modulus of Martensite (E_M_) and parameter measuring the difference between material responses in tension and compression (α).”

#### Graft

Uniaxial tensile tests under displacement control until rupture (0.02 mm/s rate, with Bose EnduraTEC 3200 – Bose Corporation, MN, USA) were carried out on graft samples cut from a Valiant Captivia stent-graft. The analyzed polyethylene terephthalate (PET) revealed a linear elastic behaviour in tension with a resultant Young’s modulus of 1080 MPa in the longitudinal direction^31^ (Figure 2c). For the simulation, PET was modelled with a linear isotropic elastic fabric material where the stiffness in compression was set to zero as the graft offers negligible resistance to compression. The Poisson’s ratio was set to 0.35.

### Crimping Test on the complete Stent-Graft Model

The stent-graft models were built by assembling the rings with the graft. The graft was discretized with 3-nodes membrane elements. 22,320, 35,136 and 23,272 elements were present in the graft part for devices A, B and C, respectively. The average element size was 1 mm: it was chosen to be consistent with the position of the sutures in the real device to create a merged node connection between the graft and stent elements (Figure 1C). The stent-graft presents a stent pre-deformation in the sutured configuration which must be included in the numerical model. The pre-stress simulation consisted of moving the nodes from the stress-free configuration to the sutured one by imposing a displacement boundary condition. Then, tied contacts between stent rings and the graft were activated to let the device assume the final configuration.

The crimping tests on the complete stent-grafts A and B were used for the validation of the device models. In particular, starting from the initial diameter, each endograft was crimped up to a diameter of 10 mm and then released.

The numerical simulation setup reflected the one described in the previous paragraph (4.1.1). Penalty contacts between the planes and stent and graft were introduced with a friction value of 0.3 for the stent and 0.1 for the graft. Also, penalty self-contacts were introduced between the stent struts to prevent penetration. After a sensitivity analysis, a damping factor of 1 s^−1^ for the stent and 0.1 s^−1^ for the graft were adopted.

### Deployment in an Idealized Aorta and Procedure Validation

#### Simulation Setting

**Figure 3:**
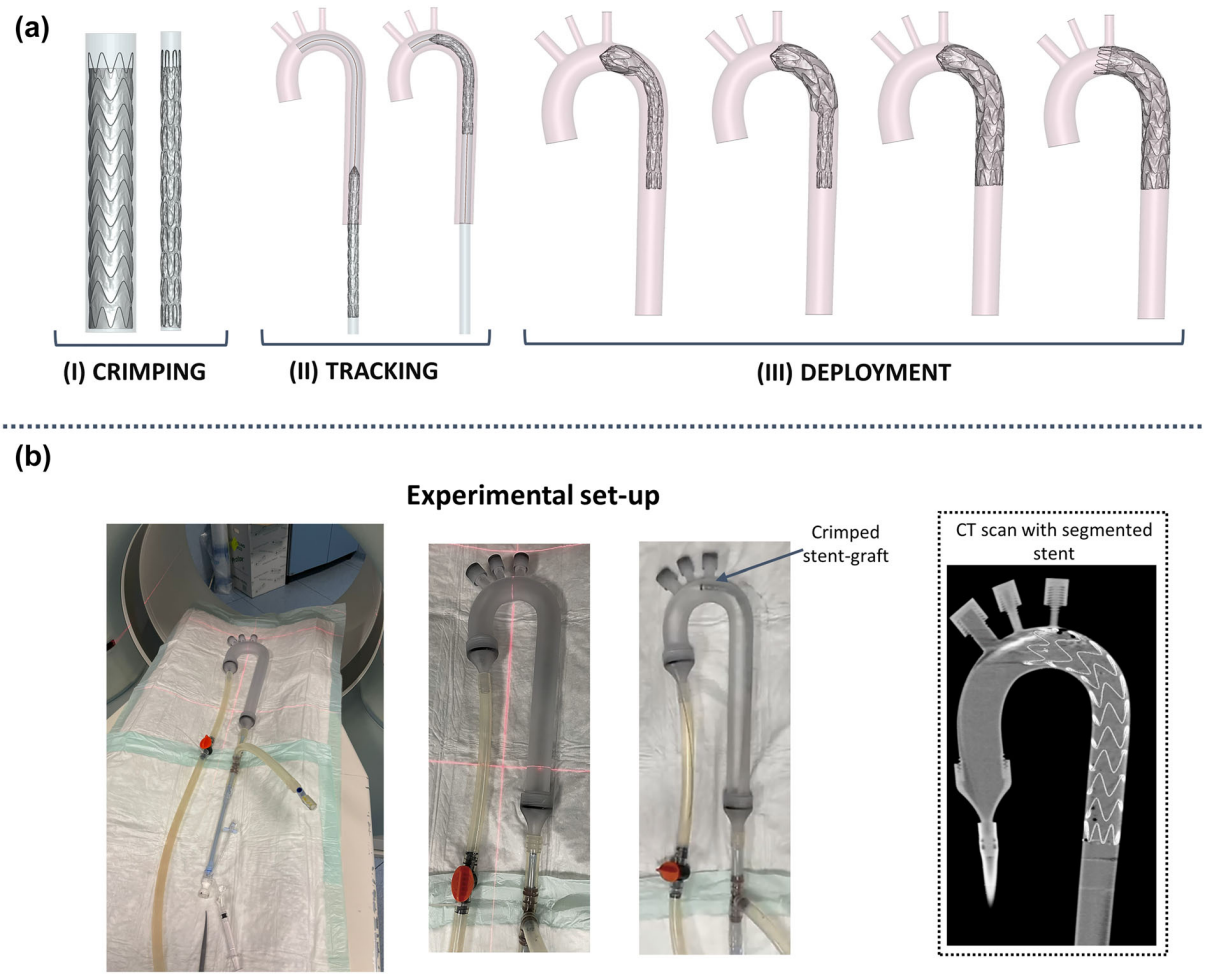
(a) Steps of the TEVAR simulation: (I) stent-graft crimping inside a catheter, (II) stent-graft tracking and (III) stent-graft deployment; (b) Experimental set-up to acquire CT images of the deployment procedure and detail of the final CT scan with the segmented stent.

A CAD aortic model with physiological anatomy^25^ was created and adopted to reproduce the TEVAR procedure. It was discretized with triangular rigid elements with an element size of 1.2 mm (total of 53,890 elements and 27,153 nodes). The aorta had a diameter of 30 mm in the proximal landing zone, thus the Valiant Captivia 34 × 34 × 200 size stent-graft (device C) was chosen, as suggested by the Valiant Captivia instructions for use.^38^ The stent-graft initial configuration, as well as the stress/strain distribution, were imported from the pre-stress procedure. A new tracking method to reproduce the clinical procedure was proposed and the numerical simulation comprised 3 steps,^23^ as reported in Figure 3a.

(I) *Stent-graft crimping*– the pre-stressed stent-graft was crimped in a catheter and penalty contacts with soft formulation between the stent-graft and crimping catheter were introduced with a friction coefficient of 0.1.

(II) *Stent-graft tracking* – the stent-graft was displaced along the aortic centerline until the proximal landing zone was reached. Penalty contacts with soft formulation were imposed between the stent-graft and the catheter without friction.

(III) *Stent-graft gradual deployment* – the stent-graft was deployed from a proximal toward a distal region by gradually unsheathing the catheter. The main body was firstly gradually released and then the proximal free flow ring, as happens in the real scenario with the tip capture mechanism.^38^ In this step, soft penalty contacts were introduced between the stent-graft and the aorta with a friction coefficient of 0.1.

During the simulation damping factors of 1 s^−1^ and 0.5 s^−1^ were imposed on the stent and the graft, respectively. The timestep was set to 0.001 ms (with mass scaling technique).

#### CT Scans and Comparison

The stent-graft configuration obtained from the simulation was compared with a real scenario to validate the numerical results. In particular, the same aorta CAD model was 3D-printed using Material Jetting technology (Stratasys J750 Digital Anatomy) and a rigid transparent photopolymer (Stratasys VeroClear RGD810), featuring the mechanical and physical properties reported in the Appendix. Threaded caps were designed to close the outflow at super-aortic branches. Two threaded ½ tube connectors were designed to connect the aortic root and the abdominal aorta to the tubing system. Both caps and connectors were 3D-printed using VeroClear along with integrated sealings 3D-printed with a deformable photopolymer (Agilus30 Black). The vessel was closed filled with water at 37 °C to reproduce temperature physiological conditions. Device C was experimentally implanted in the model and the deployment was performed under CT scan (Somatom Definition, Siemens Healthcare s.r.l.). Four scans at different timing during the deployment were acquired: first stent ring releasing, half body releasing, main body fully deployment and free flow ring releasing. Using the open-source software VMTK (Orobix s.r.l.), the stent was segmented from each CT acquisition and compared with the simulation results. The experimental set-up is reported in Figure 3b. A qualitative comparison of the position of the device was managed by superimposing the segmented stent on the simulation results. Then, using a Matlab code, a spline was fitted into the apexes of each stent ring and the area enclosed by the spline was evaluated to quantify each stent ring opening area (OA). The OA percentage error between the experiment and the numerical simulation was computed as well.^18,19,30^ In addition, the maximum absolute distance between the simulation and segmentation for each stent strut was calculated to evaluate the stent strut alignment.

### Stent-Graft Model Verification Analysis and Literature Comparison

For the verification process of the stent-graft model, a sensitivity study on the impact of the modeling features was carried out. The list of the performed simulations is reported in Table 3. The newly proposed tracking method (Figure 3A) and the literature “virtual catheter” method^4,5,18,19,32^ were compared. With the literature approach, the stent-graft was simultaneously crimped and morphed by a catheter until the vessel centerline was reached. Then, the catheter was removed to let the device expand: the stent-graft was deployed at once and the free flow ring was not released as last, but simultaneously with all the stent struts. In particular, simulations with Nitinol modeled as linear elastic material (*E* = *E*_*A*_ = *57,500 MPa*^13,16,21,29^) or with PET as linear elastic material (*E* = *1.84 MPa*^18,20,33^ and *E* = *1.84 GPa*^24^) were tested as well as the effect of the absence of the stent pre-stress.^18,19,33^ Also, to investigate the stent-graft releasing phase, a condition of modified “virtual catheter” method was studied where the stent-graft was deployed gradually.

**Table 3:** List of the simulations carried out for the verification process.

ID		Deploy method	Nitinol	Stent pre-stress	PET
01	*Nitinol linear elastic*	Tracking*	Linear elastic E = E_A_ = 57,500 MPa^14,16,22,31^	Yes	Fabric*
02	*PET linear elastic – 1.84 MPa*	Tracking*	Super-elastic*	Yes	Linear elastic E = 1.84MPa^18,20,33^
03	*PET linear elastic – 1.84 GPa*	Tracking*	Super-elastic*	Yes	Linear elastic E = 1.84GPa^24^
04	*Without stent pre-stress*	Tracking*	Super-elastic*	No^18,19,33^	Fabric*
05	*Virtual catheter method—fabric*	Virtual Catheter^4,5,18,19,32^	Super-elastic*	Yes	Fabric*
06	*Virtual catheter method – Linear elastic 1.84 MPa*	Virtual Catheter^4,5,18,19,32^	Super-elastic*	Yes	Linear elastic E = 1.84MPa^18,20,33^
07	*Virtual catheter method—Linear elastic 1.84 GPa*	Virtual Catheter^4,5,18,19,32^	Super-elastic*	Yes	Linear elastic E = 1.84GPa^24^
08	*Virtual catheter method modified*	Virtual Catheter^4,5,18,19,32^ + gradual release	Super-elastic*	Yes	Fabric*

*New tracking method and material parameters are features of the present study

The results of the different simulations were analysed in terms of simulation correctly running (positive or negative whether the simulation ended correctly or not) and, in the case of ended simulation, in terms of the opening area and deployed stent-graft length with respect to the CT configuration.

## Results

### Validation with Crimping Test on the Single Ring and on the Complete Stent-Graft Model

**Figure 4:**
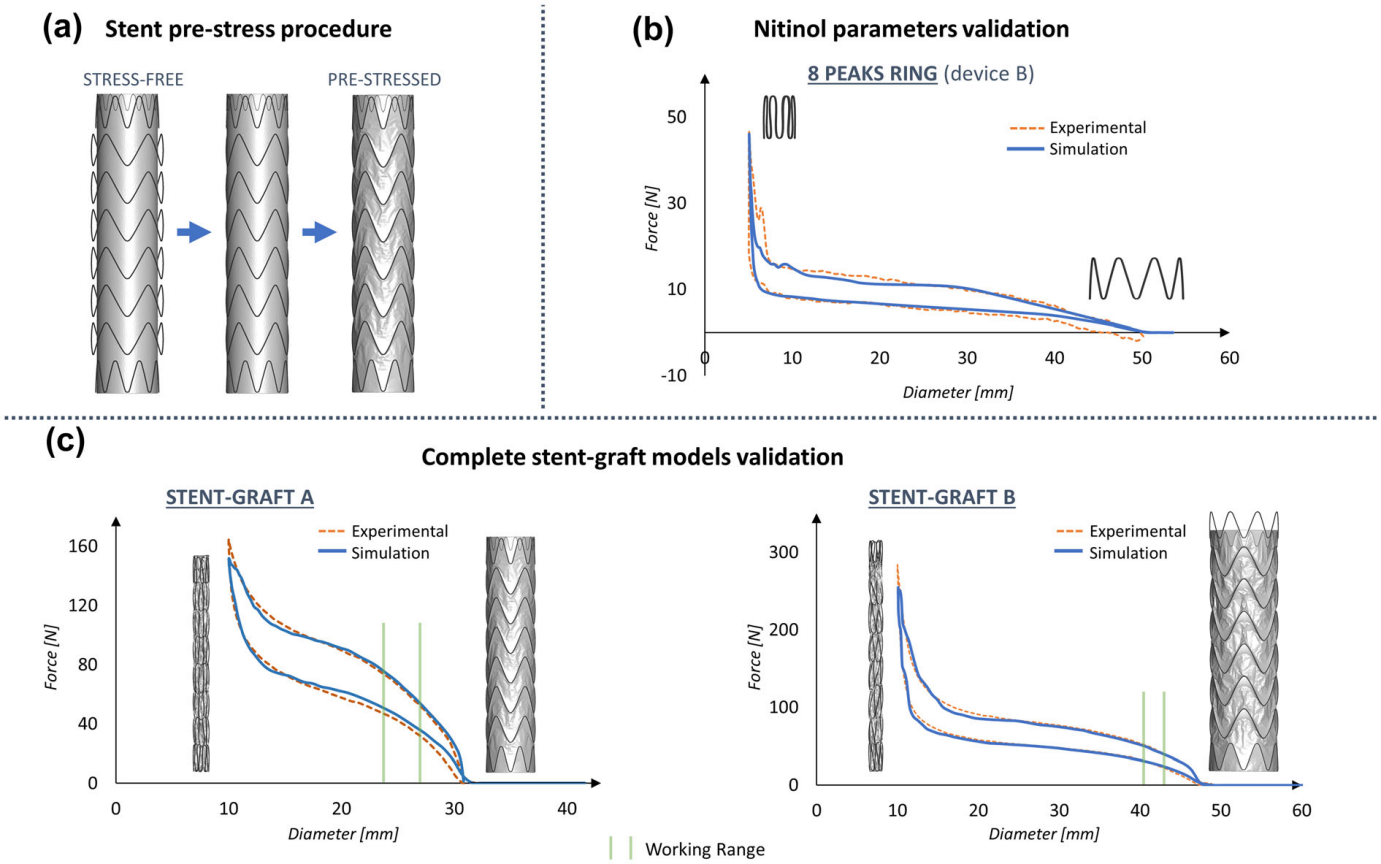
(a) Stent pre-stress procedure (reported only for device A); (b) Nitinol parameters validation on the 8-peaks ring of device B; (c) Complete stent-grafts A and B validation results. The working range of the stent-grafts is highlighted in green.

The stent pre-stress procedure is reported in Figure 4a. The material parameters obtained from the single ring and PET tests (Figure 2) were validated by comparing the results of both the crimping/release tests performed on the 8 peaks stent ring of device B and the complete stent-grafts A and B (Figures 4b and 4c). The finite element curve overlaps the experimental data resulting in a proper validation of the stent-graft model. The error between the experimental and calibrated curves in the working range of the stent-grafts is 2.6 ± 1% for device A and 1.54 ± 0.24% for device B on the loading curve path, and 4 ± 1.8% for device A and 1.9 ± 1.8% for device B on the unloading path.

### Deployment in an Idealized Aorta and Validation Procedure

**Figure 5:**
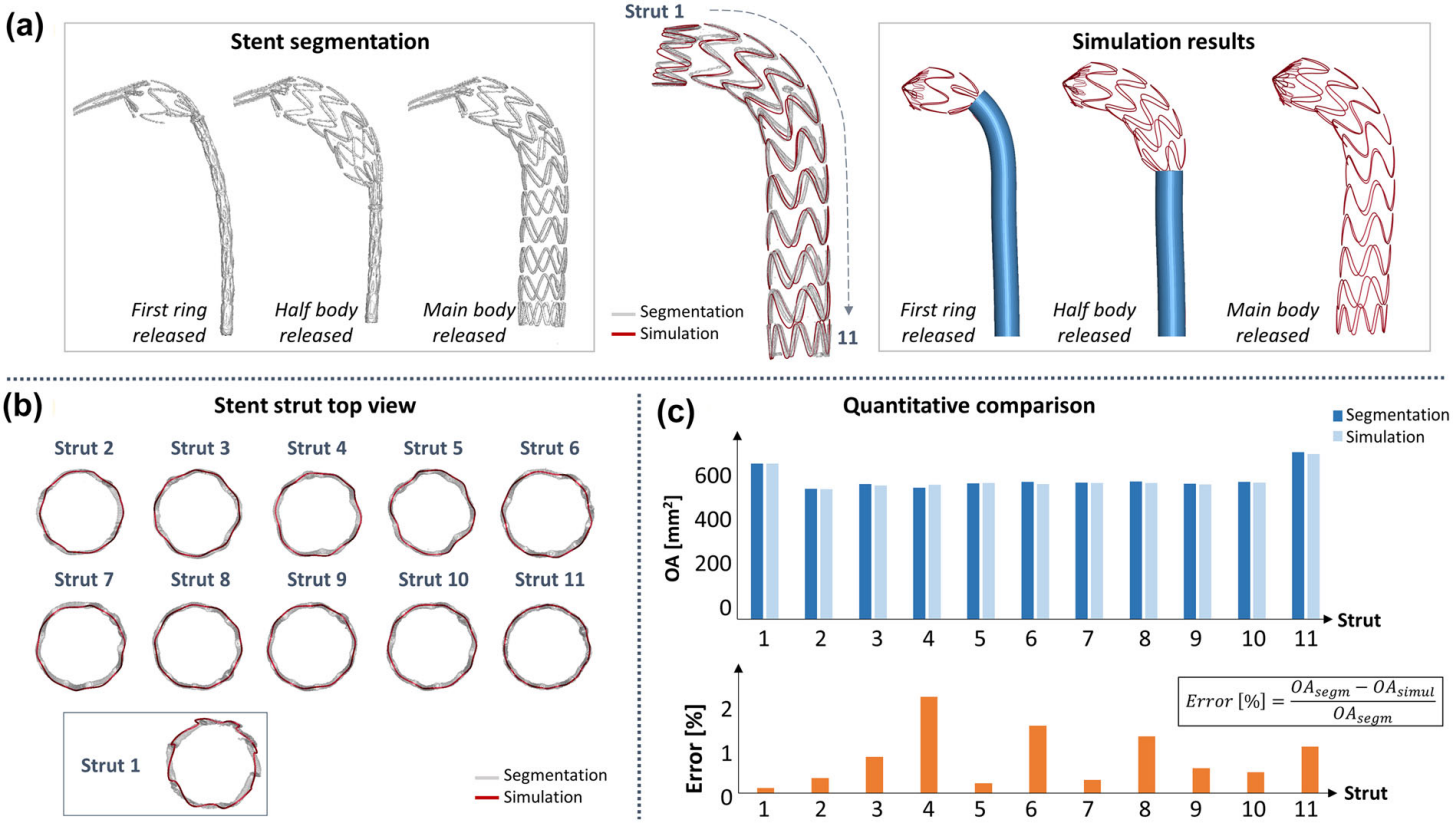
(a) Quantitative comparison: segmentation and numerical simulation results at different deployment timings. The final configurations from CT (grey) and simulation (red) are overlapped; (b) Stent strut qualitative comparison with the top view representation; (c) Quantitative comparison: top: opening area for the CT and simulation final configuration at each stent strut (1 = proximal, 11 = distal); bottom: opening area (OA) % error for each stent strut.

Figure 5a shows the stent segmentation (the graft was not visible from CT images) and numerical simulation results at different steps during the deployment. At these different timings, the simulation results were qualitatively compared with the CT to correctly set-up the simulation. Also, the qualitative comparison is displayed by superimposing the CT and numerical results only for the final deployed configuration. In Figure 5b, the top view for each stent ring is shown, in which the two configurations are overlapped. The final configuration is properly detected by the numerical model in terms of stent ring expansion and global stent-graft length. The maximum distance between the simulation and segmentation for each strut was calculated and compared to the strut diameter: the error was found to be lower than 4%.

The OA of each stent strut (Figure 5c) was evaluated for both deployed configurations and the absolute value of the OA percentage error is reported for each stent strut. The error is found to be always below 2.5% (0.92% on average) and it is almost negligible in the free flow strut 1.

### Stent-Graft Model Sensitivity Analysis and Literature Comparison

**Figure 6:**
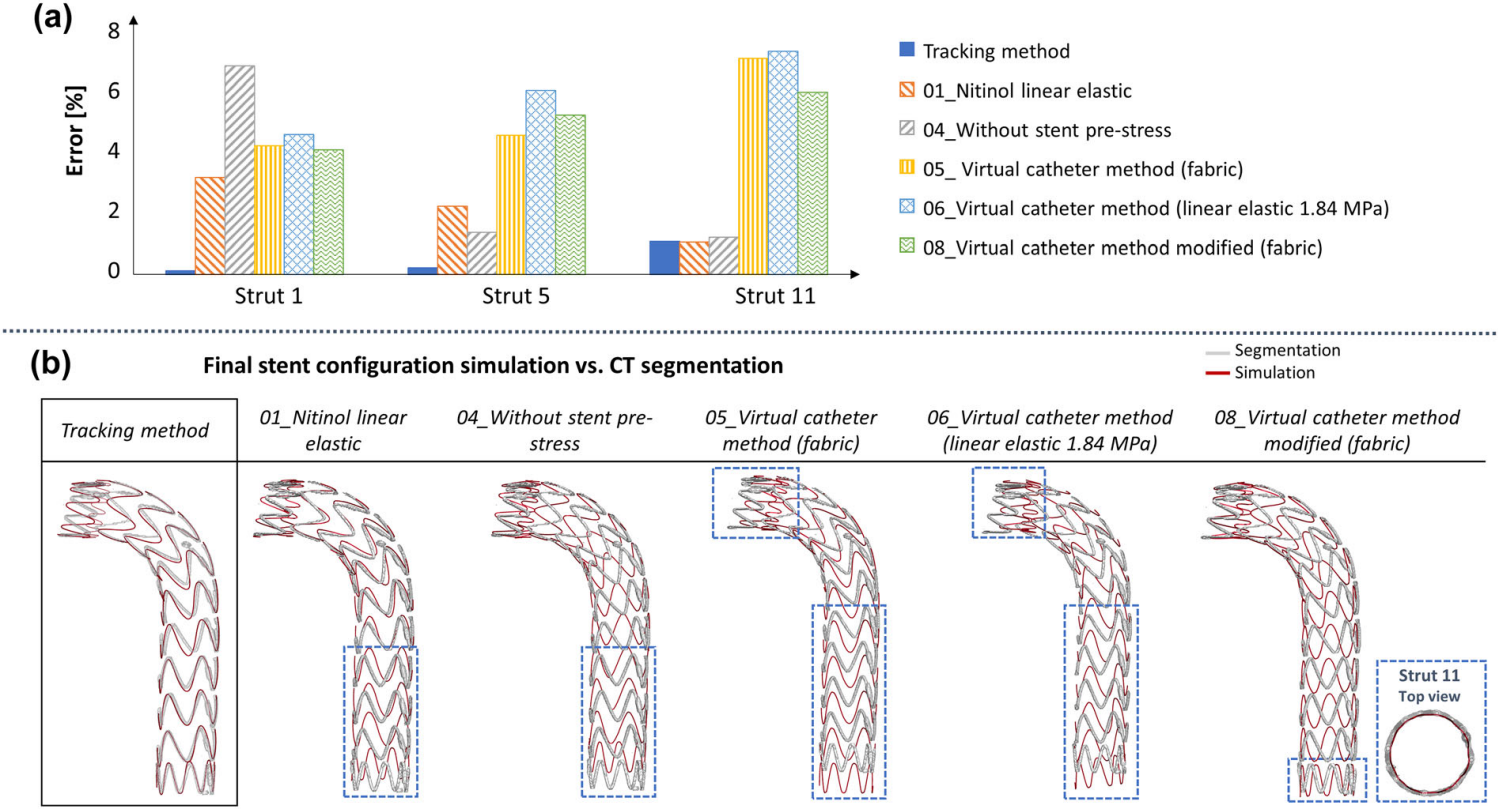
(a) Verification simulations opening area (OA) errors [%] computed with respect to the CT configuration for the positive outcome simulations. For sake of readiness only the proximal free flow (strut 1), a central (strut 5) and the distal (strut 11) struts errors are reported; (b) The final stent configuration for the positive outcome simulations (grey) overlapped with the CT segmentation configuration (red). The discrepancies in the stent-graft length are highlighted in blue.

Table 4 shows the list of the simulation results for the verification analysis in terms of average opening area and % error on the OA. Simulations with Nitinol as linear elastic (01), without stent pre-stress (04) and with the ‘virtual catheter’ deployment method both with graft as fabric (05, 08) and linear elastic with E = 1.84 MPa (06) ended correctly. The case with PET as linear elastic with both E = 1.84 MPa (02) and E = 1.84 GPa (03) and the tracking method, and with only E = 1.84 GPa (07) and the “virtual catheter” method ended with a fatal error due to extremely high and bad deformations. In simulation 01, the OA error is always below 4%; the strut 1 (free flow) opening area is underestimated slightly with respect to the CT and the reference simulation (Figure 6a). In simulation 04, the stent-pre-stress was neglected: the discrepancies with the CT are evident mainly at strut 1 in which the error is maximum (OA of 539 mm^2^ vs. CT OA of 579 mm^2^) (Figure 6a). In simulations 05 and 06 with the “virtual catheter” approach, the OA error is higher with respect to the tracking method, especially in the distal rings (Figure 6a). In simulation 08, the “virtual catheter” approach is modified including the gradual deployment of the stent-graft: regarding the OA error, similar considerations to simulations 05 and 06 can be derived.

**Table 4:** Verification simulations with the outcome, opening area (OA) and OA % error with respect to CT.

ID		Outcome	OA [mm^2^] Average ± std dev smin; max]	Error [%] Average ± std dev [min; max]
–	*CT segmentation*	–	521.51 ± 41.18 *[485.013; 621.704]*	–
–	*Tracking method*	–	518.84 ± 40.20 *[483.33; 615.0272]*	0.92% ± 0.64% *[0.12%; 2.23%]*
01	*Nitinol linear elastic*	Positive	511.71 ± 40.16 *[482.33; 615.19]*	2.26% ± 1.15% *[0.3%; 3.59%]*
02	*PET linear elastic – 1.84 MPa*	Negative	–	–
03	*PET linear elastic – 1.84 GPa*	Negative	–	–
04	*Without stent pre-stress*	Positive	522.44 ± 32.65 *[498.37; 614.1828]*	2.37% ± 1.89% *[0.30%; 6.83%]*
05	*Virtual catheter method—fabric*	Positive	488.89 ± 38.99 *[463.98; 577.703]*	6.11% ± 1.98% *[2.93%; 9.15%]*
06	*Virtual catheter method – Linear elastic 1.84 MPa*	Positive	484.35 ± 40.36 *[453.41; 576.21]*	6.99% ± 2.34% *[3.54%; 11.22%]*
07	*Virtual catheter method—Linear elastic 1.84 GPa*	Negative	–	–
08	*Virtual catheter method modified*	Positive	489.06 ± 40.96 *[462.34; 584.59]*	6.10% ± 1.81% *[3.15%; 8.95%]*

The average ± standard deviation and the maximum and minimum values are reported. “Tracking method” OA and OA % error are referred to the validated model

By qualitatively analysing the deployed stent-graft length (Figure 6b), in all the investigated literature cases, except for simulation 08, discrepancies were found with respect to the segmented stent.

## Discussion

When performing numerical simulations, the reliability and truthfulness of numerical models are of primary importance and the verification and validation (V&V) process is crucial. In this sense, the synergy between numerical and experimental studies is fundamental.^3,28,40^ This is particularly true for stent-graft simulations where different modelling and numerical strategies are adopted in the literature. In this context, investigating the mechanical behaviour of a commercial stent-graft by assigning the correct material properties to each component is necessary for the reliability of the numerical model itself.

In our study, the material parameters of a commercial stent-raft are deeply investigated, calibrated and validated based on experimental tests and compared with literature values. In fact, from the material modeling perspective, no consensus is present in the literature on the stent-graft material properties. Nitinol is modelled either as superelastic or it is simplified as linear elastic with a Young’s modulus equal to austenite.^12,15,21,29^ In addition, if modelled as superelastic, the Nitinol parameters are different among the different literature works. On the other hand, PET is described as a linear elastic material, but the Young’s modulus varies from 1.84 MPa^19,20,33^ to 1.84 GPa.^24^ Furthermore, in the work by Demanget *et al.*,^12^ a more elaborated linear elastic orthotropic material was proposed to differently model the Young’s modulus in the warp (225 MPa) and weft (1 GPa) directions. As regards the mechanical behaviour, the radial force is a fundamental indicator of the stent-graft effective fixation to the aortic wall^36,41^ and it is mainly investigated through ad-hoc crimping tests. For this reason, reproducing in silico a realistic behaviour obtained with crimping experimental tests^10,8,42^ is relevant for successfully replicating the TEVAR procedure. De Bock *et al.*^10^ detailed the mechanical behaviour of commercial abdominal stent-grafts, and the Nitinol and PET material parameters were calibrated on plate compression and crimping tests on the whole device. Similarly, Concannon *et al.*^8^ performed crimping/release experimental tests on a single Valiant Captivia stent strut without graft to calibrate the Nitinol parameters.

In our work, ad-hoc crimping experimental tests were performed both on single stent struts and on the stent-graft and were used for both material calibration and device model validation purposes. In addition, tests were carried out at 37 °C to guarantee the working condition of the stent-graft. Our final Nitinol calibrated material parameters of the single stent ring accounted also for the ring deformed shape since they were not directly derived from Nitinol wires. Also, an innovative PET fabric formulation was proposed to simulate the fabric behavior with no resistance to compression. The final PET material parameters include both the polymer woven stiffness and the presence of the sutures. In fact, the suture points were not directly modelled, but they were considered in the node-to-node connections between the stent and graft elements. The material parameters we identified allowed to simulate both the Nitinol and stent-graft model in the working ranges, 25–27 mm for the ring from device A and 41-42 mm for device B, since the obtained curves properly fit the experimental data (errors below 4% in the stent-grafts working ranges).

Another key factor that highly affects the global behaviour of a stent-graft, is the stent pre-stress. Among the recent literature, few works dealing with stent-grafts introduced this feature in a FE model. Roy *et al.*^34^ considered the stent pre-deformation by doubling the stent Young’s modulus. Derycke *et al.*^13^ included the stent pre-stress in the model for a custom-made thoracic stent-graft, but the Nitinol was simplified as linear elastic. Perin *et al.*^30^ included the stent pre-stress in a commercial stent-graft model for abdominal aneurysm adopting the superelastic Nitinol formulation. Recently, Hemmler *et al.*^17^ detailed a general stent-graft pre-deformation procedure for a Stainless Steel stent for the abdominal aorta and investigated the influence of the pre-stress on the vessel wall. The work presented by Concannon *et al.*^8^ studied the mechanical behaviour of a stent-graft including the stent pre-stress. However, they only calibrate Nitinol material parameters on one stent ring and simplified the complete stent-graft model by creating a homogenized model. To the best of the authors’ knowledge, our work is the first one that develops thoracic stent-graft numerical models accounting for the stent pre-stress in the stent-graft assembly phase and validating the numerical results on experimental data.

Concerning the deployment simulation, a new tracking methodology for replicating the TEVAR procedure was developed and validated with experimental results. The numerical simulation truthfully reproduced the deployment mechanism of a stent-graft also replicating the tip capture mechanism.^38^ Also, with this new method, the stent-graft follows the vessel curvature during the tracking phase, as happens in the real scenario when the device is inserted into the patient. The use of the FE method to reproduce the TEVAR technique is supported by many literature studies^5,13,16,18,19,33^ which performed validation on the final stent-graft configuration by comparing the simulation results with stent segmented from patient-specific CT images. However, the V&V process on the TEVAR procedure simulations is not always done to assess the credibility of the in silico results.^37^ To the best of the authors’ knowledge, the finite element TEVAR simulations were never compared at different timing during the deployment with an ad-hoc experimental setup. In our framework, the aorta was 3D printed with a rigid material^21^ to prevent numerical uncertainties related to the arterial wall mechanical properties. Furthermore, the vessel was closed and filled with water at 37 °C. The comparison between the numerical result and the segmented stent from CT images showed a good agreement both in terms of qualitative overlapping and quantitative area evaluation of the two final positions. The opening area error is small (always below 2.5%) and almost negligible in the proximal strut where incorrect sealing and malapposition may compromise the outcome of the TEVAR procedure. Moreover, the OA error resulting from the proposed tracking method is found to be much smaller than OA errors found in literature studies which range from 5 to 30%.^18,19,30^ The opening area is a fundamental indicator of the stent-graft anchoring to the aortic wall. If the stent struts are well attached to the vessel, the possibility of developing stent-graft related complications such as bird beak, endoleak or migration is reduced.^26^

Since the goal of the study was to develop high fidelity FE stent-graft models, it was necessary to perform a verification analysis on the modeling choices. By exploiting the most common literature adopted options, the OA error with respect to the CT configuration was always higher than the proposed tracking model. Considering the Nitinol as a linear elastic material, a strong limitation is introduced in the stent strut behaviour and the stress and strain distributions developed during the procedure. When PET was simulated as linear elastic, the tracking simulation failed due to extremely high and bad deformations. The results of the simulation without stent pre-stress confirmed that the pre-stress plays a significant role in the stent strut opening area, especially in the free flow ring and proximal stent struts. In the literature, the TEVAR procedure is mainly modelled by following the “virtual catheter” method.^4,18,33^ With this approach, the simulation works correctly only if PET is modelled as fabric or, despite is not realistic, as linear elastic with E = 1.84 MPa.^19,20,33^ From our analysis, the “virtual catheter” method with fabric introduces errors in the stent struts OA (underestimated with respect to the CT). Also, the deployed stent-graft was longer with respect to the CT because the gradual deployment was not considered. In fact, with the gradual expansion, the stent-graft shortens a bit its length every time each stent strut is released. This is verified with the modified “virtual catheter” approach in which the deployed stent-graft length was captured because the device was deployed gradually.

The study is not free from limitations like the adoption of an idealized and rigid aortic model or the consideration of only one design of stent-graft. Also, the effect of the presence of the guidewire was neglected. However, the simplifications are reasonable since the focus of the study is on the development of high-fidelity stent-graft models and realistic modeling of the TEVAR procedure, which can be followed for any specific stent-graft design. Indeed, the obtained results can be generalized to any size of a stent-graft and patient-specific deformable anatomies reconstructed from clinical images can be easily adopted for pre-procedural planning.
